# Development of a new score for early mortality prediction in trauma ICU patients: RETRASCORE

**DOI:** 10.1186/s13054-021-03845-6

**Published:** 2021-12-07

**Authors:** Luis Serviá, Juan Antonio Llompart-Pou, Mario Chico-Fernández, Neus Montserrat, Mariona Badia, Jesús Abelardo Barea-Mendoza, María Ángeles Ballesteros-Sanz, Javier Trujillano, the Neurointensive Care and Trauma Working Group of the Spanish Society of Intensive Care Medicine (SEMICYUC)

**Affiliations:** 1 Servei de Medicina Intensiva, Hospital Universitari Arnau de Vilanova, Universitat de Lleida, IRBLleida, Lleida, Spain; 2 Servei de Medicina Intensiva, Hospital Universitari Son Espases, Institut d’Investigació Sanitària Illes Balears (IdISBa), Palma de Mallorca, Spain; 3 UCI de Trauma y Emergencias, Servicio de Medicina Intensiva, Hospital Universitario 12 de Octubre, Madrid, Spain; 4 Servicio de Medicina Intensiva, Hospital Universitario Marqués de Valdecilla, Santander, Spain; 5 Intensive Care Unit, Hospital Universitario Arnau de Vilanova, Avda Rovira Roure 80, 25198 Lleida, Spain

**Keywords:** Trauma and injury severity score, Intensive care unit, Mortality, Prognostic scoring systems

## Abstract

**Background:**

Severity scores are commonly used for outcome adjustment and benchmarking of trauma care provided. No specific models performed only with critically ill patients are available. Our objective was to develop a new score for early mortality prediction in trauma ICU patients.

**Methods:**

This is a retrospective study using the Spanish Trauma ICU registry (RETRAUCI) 2015–2019. Patients were divided and analysed into the derivation (2015–2017) and validation sets (2018–2019). We used as candidate variables to be associated with mortality those available in RETRAUCI that could be collected in the first 24 h after ICU admission. Using logistic regression methodology, a simple score (RETRASCORE) was created with points assigned to each selected variable. The performance of the model was carried out according to global measures, discrimination and calibration.

**Results:**

The analysis included 9465 patients: derivation set 5976 and validation set 3489. Thirty-day mortality was 12.2%. The predicted probability of 30-day mortality was determined by the following equation: 1/(1 + exp (− *y*)), where *y* = 0.598 (Age 50–65) + 1.239 (Age 66–75) + 2.198 (Age > 75) + 0.349 (PRECOAG) + 0.336 (Pre-hospital intubation) + 0.662 (High-risk mechanism) + 0.950 (unilateral mydriasis) + 3.217 (bilateral mydriasis) + 0.841 (Glasgow ≤ 8) + 0.495 (MAIS-Head) − 0.271 (MAIS-Thorax) + 1.148 (Haemodynamic failure) + 0.708 (Respiratory failure) + 0.567 (Coagulopathy) + 0.580 (Mechanical ventilation) + 0.452 (Massive haemorrhage) − 5.432. The AUROC was 0.913 (0.903–0.923) in the derivation set and 0.929 (0.918–0.940) in the validation set.

**Conclusions:**

The newly developed RETRASCORE is an early, easy-to-calculate and specific score to predict in-hospital mortality in trauma ICU patients. Although it has achieved adequate internal validation, it must be externally validated.

**Supplementary Information:**

The online version contains supplementary material available at 10.1186/s13054-021-03845-6.

## Background

Severe trauma remains the leading cause of mortality and disability in young adults [1]. In this setting, trauma registries provide relevant information in terms of benchmarking of the care provided and, therefore, constitute a relevant contribution to quality assessment and scientific research in an area where classical randomized trials are difficult to perform [2–4]. To this purpose, the *Injury Severity Score* (ISS) has been the most commonly used score to assess severity of trauma [5]. Several years later, the *Trauma and Injury Severity Score (TRISS)* became the most frequently used tool for outcome adjustment and benchmarking in worldwide trauma registries [6].

More recently, The TraumaRegister DGU™ (TR-DGU) developed the Revised Injury Severity Classification (RISC) score [7] and its updated version in 2014 [8] for outcome adjustment, achieving an astonishing AUC 0.95 in the derivation and validation datasets in terms of mortality prediction, with appropriate precision and calibration [8]. However, these scores can be applied to the general trauma patients and, therefore, are not specific for the trauma ICU patients, in whom the physiological consequences of trauma itself play a major role in outcomes. Indeed, intensive care units (ICUs) commonly use severity scores such as the *Acute Physiology and Chronic Health Evaluation* (APACHE II), the *Simplified Acute Physiologic Score* (SAPS II) or the *Mortality Probability Models* (MPM II), which take into consideration age, comorbidities and the physiological burden of critical illness rather than anatomic considerations [9].

Due to the lack of a specific trauma ICU score and by using data from the *Spanish Trauma ICU registry* (RETRAUCI), our objective was to develop a new score for early mortality prediction in trauma ICU patients.

## Methods

This is a retrospective study that aims to develop and validate a mortality prognostic model with variables included in the RETRAUCI project. RETRAUCI is an observational, prospective and multicentre nationwide registry that currently includes 52 ICUs in Spain. It has the endorsement of the Neurointensive Care and Trauma Working Group of the Spanish Society of Intensive Care Medicine (SEMICYUC) and currently operates in a web-based electronic format (www.retrauci.org). Additional file 1 shows the screenshots of the web application and the list of variables collected in RETRAUCI. The records for the years 2015–2019 were used. To achieve internal validation, the total records were divided into two sets: derivation set (2015–2017) and validation set (2018–2019). It is a study with complete-case analysis with temporal validation. The development of the models was carried out following the recommendations established in the Transparency Reporting of a multivariable prediction model for Individual Prognosis or Diagnosis (TRIPOD) initiative [10].

Ethics Committee approval for the registry was obtained (Hospital Universitario 12 de Octubre, Madrid: 12/209). Due to the retrospective analysis of de-identified collected data, informed consent was not obtained for this study.

### Patients and variables included

Patients were managed according to the *Advanced Trauma Life Support* principles. Data on epidemiology, acute management in the pre-hospital and in-hospital settings, type and severity of injury, resources utilization, complications and outcomes were recorded. We only excluded patients with missing data about in-hospital mortality. Possible candidate variables have been selected according to clinical, bibliographic and availability criteria in the RETRAUCI database. The candidate variables must also be available within the first 24 h of admission to the ICU. The variables entered were then analysed according to different categories:

Patient-related variables included sex, age and prior antiplatelet/anticoagulant treatment. Sex was treated as a dichotomic variable (male/female), age was distributed in four categories (less than 50 years, 50 to 65 years, 66 to 75 years and older than 75 years) and if the patient was on chronic treatment with antiplatelets or anticoagulants he/she was considered to have prior coagulation alteration (PRECOAG) [11].

Pre-hospital care variables included pre-hospital medical attention, pre-hospital intubation and mechanism of trauma, which differentiates penetrating vs. non-penetrating types. Additionally, we coded as a high-risk mechanism trauma those mechanisms with associated mortality higher than 20%. This category included gunshot wounds, pedestrian falls, accidental falls, suicidal precipitation and those considered as unknown mechanism.

Physiological variables: pupillary size and reactivity (normal, unilateral mydriasis and bilateral mydriasis), and score of the Glasgow Coma Scale (absolute score and percentage of patients with ≤ 8 points).

Anatomical variables describing the severity of injuries according to the Abbreviated Injury Scale (AIS) were considered. The AIS ranges from 0 to 6, with 0 indicating no involvement and 6 indicating maximum involvement [12]. A major organic involvement was considered with a score of 3 or higher (MAIS) in any of the following six anatomical areas: head (MAIS-Head), thorax (MAIS-Thorax), abdomen (MAIS-Abdomen), upper extremity (MAIS-Ext Upper), lower extremity (MAIS-Ext Lower) and external and thermal injuries (MAIS-External).

Organ failure-related variables were also considered: haemodynamic failure indicated by systolic blood pressure lower than 90 mmHg requiring the administration of volume, blood products and vasoactive support; respiratory failure, indicated by pO2/FiO2 below 200; and coagulopathy, indicated by the prolongation of prothrombin and activated partial thromboplastin times in > 1.5 times the control or by levels of fibrinogen < 150 mg/dL or thrombocytopenia < 100,000/µL in the determination of the first 24 h [13].

Treatment variables included the need of mechanical ventilation and the activation of the massive transfusion protocol because of a massive haemorrhage [14].

### Outcome definition

The outcome variable was defined as 30-day mortality after trauma. Patients who were discharged from the hospital alive before 30 days after trauma were assumed to have survived for at least 30 days.

The ICU length of stay (LOS) was also collected to compare the derivation and validation sets. The probability of death (1-probability of survival) according to the TRISS score was used as a comparison model [15].

### Statistical analysis

The sample size calculation helped us to verify that there were enough records for the development and validation of the model. For each possible factor, 10 deaths are needed. With a mortality of 12% and 20 variables as potential risk factors, at least (20 × 10/0.12) = 1666 records are needed for the derivation and validation sets [16].

Categorical variables were described as percentage and continuous variables as median (interquartile range), as they did not follow a normal distribution (Kolmogorov–Smirnov test). For the comparison between the groups derivation–validation and survivors–non-survivors, the Mann–Whitney test was used for continuous variables and the Chi-square test for categorical variables. A *p* < 0.05 was considered statistically significant.

In the derivation set, a multivariable logistic regression model was used to determine predictors for 30-day mortality. We use the LASSO (Least Absolute Shrinkage and Selection Operator) logistic regression algorithm in order to obtain a subset of predictor variables from the 20 candidate variables. The LASSO algorithm can select from the set of candidate variables that achieve greater importance once regularized. The LASSO algorithm finds the variables that contribute the least in the logistic regression model and forces them to have coefficients equal to zero. In this way, only the significant variables will be part of the final model [17]. This subset of predictor variables was used to carry out the logistic regression model and the odds ratios with their 95% confidence intervals, and the *β*-coefficients of each factor were calculated.

To check the stability in the selection of variables and in the calculation of the coefficients of the logistic regression, an analysis with random partitions of the derivation and validation sets was carried out. One hundred sets of derivation have been created randomly with the same number of records as those used in the temporal validation (*n* = 5976). The LASSO variable selection methodology has been applied to each of these sets, and the corresponding logistic regression coefficients have been calculated. These results were compared with those obtained in the temporal validation.

Internal validity and adjustment for overfitting of the model were performed with a bootstrapping procedure. One thousand bootstrap samples were drawn from the derivation set. A shrinkage factor that multiplied the *β*-coefficients of the predictive factors and made them adjusted was calculated. These *β*-coefficients were used to calculate the individual probability of death in the derivation and validation sets.

A simple score (RETRASCORE) was developed based on predictors that were associated with 30-day mortality in the multivariate analysis. Score points were defined multiplying the regressions *β*-coefficients by 2 and rounded them to the nearest integer. The sum of the points is the value of the final score.

The performance of the models, logistic regression (LR), TRISS and RETRASCORE was determined in the derivation and validation sets. Global measure was used with the Brier score, discrimination measures using the area under the ROC curve (AUROC) with 95% CI, and calibration with calibration plots (the mean of the predicted probabilities was computed for each risk decile) and the calculation of the values of the fitted lines with intercept and slope with 95% CI [18].

The calculations were performed using STATA software, version 15.0 (Stata Corporation, College Station, Texas, USA) and R statistics 4.0.3 with the “glmnet” package (R Foundation for Statistical Computing, Vienna, Austria) [19].

## Results

The study group included a total of 9465 patients with complete data who were divided into a derivation set with 5976 patients and a validation set with 3489 patients. Additional file 2: Figure S1 shows the flowchart of the study. Thirty-day mortality reached 12.2%. Table 1 shows the variables potentially associated with mortality selected from the RETRAUCI database, differentiating between the derivation and validation sets. The only significant differences found in the validation set were a higher percentage of pre-hospital medical attentions (80.1 vs. 76.1%), a higher proportion of assaults (6.0 vs. 4.3%) and a lower need of mechanical ventilation (49.8 vs. 45.9%).

**Table 1:** Demographic and clinical characteristics of the whole sample and distributed by the derivation and validation sets

Variable	All *N* = 9465	Derivation *N* = 5976	Validation *N* = 3489	*p* value
Age (years)	49 (33–65)	48 (33–65)	48 (33–63)	0.183
Age (groups)				0.145
< 50	53.7	53.6	53.8	
50–65	22.8	22.3	23.7	
65–75	12.6	12.7	12.3	
> 75	11.0	11.4	10.2	
Sex (% male)	77.7	77.8	77.6	0.800
PRECOAG	13.1	13.7	12.0	0.190
Pre-hospital attention	77.6	76.1	80.1	< 0.001
Pre-hospital intubation	24.7	25.0	24.3	0.421
Type of injury				0.005
Road Traffic accident	42.8	43.2	42.2	
Fall	27.8	27.9	27.6	
Occupational accident	7.9	7.8	8.0	
Sport related	5.6	5.8	5.2	
Assault	4.9	4.3	6.0	
Self-injury	6.0	6.0	6.1	
Unknown	1.9	1.8	2.2	
High-risk mechanism	38.0	38.1	37.8	0.740
Mechanism penetrant	6.1	5.8	6.5	0.187
Pupils				0.486
Normal	88.9	88.7	89.3	
Unilateral mydriasis	6.8	7.1	6.4	
Bilateral mydriasis	4.3	4.3	4.2	
GCS	14 (9–15)	15 (9–15)	14 (9–15)	0.896
GCS ≤ 8 (%)	24.4	24.5	24.1	0.667
MAIS-Head	41.9	41.7	42.3	0.562
MAIS-Thorax	38.1	39.0	36.5	0.119
MAIS-Abdomen	12.3	12.0	12.7	0.375
MAIS-Ext Upper	2.8	2.7	3.1	0.257
MAIS-Ext Lower	17.7	17.7	17.7	0.964
MAIS-External	1.0	1.1	1.0	0.602
Haemodynamic failure	22.0	22.4	21.2	0.179
Respiratory failure	11.8	11.5	12.4	0.213
Coagulopathy	15.9	15.7	16.3	0.517
Mechanical ventilation	48.4	49.8	45.9	< 0.001
Massive haemorrhage	6.0	6.5	5.3	0.026
TRISS (% mort)	5.7 (1.7–21.4)	5.7 (1.7–21.5)	5.6 (1.6–21.3)	0.103
ICU LOS (days)	4 (2–10)	4 (2–10)	4 (2–9)	0.235
Hospital LOS (days)	12 (6–26)	12 (6–25)	11 (5–24)	0.249
30-day mortality	12.2	12.6	11.6	0.190

Values expressed as percentages or median (Interquartile range)

PRECOAG, prior treatment with antiplatelets or anticoagulants; PRE-INTUB, pre-hospital intubation; GCS, Glasgow Coma Score; AIS, Abbreviated Injury Scale; MAIS, AIS ≥ 3; MV, mechanical ventilation; TRISS, Trauma and Injury Severity Score; ICU, intensive care unit; LOS, length of stay; *p *value, calculated using Chi-square test or Mann–Whitney test

### Predictors of 30-day mortality

Table 2 shows the twenty candidate variables according to 30-day mortality in the derivation set. The LASSO regression analysis determined that 13 variables, with *β*-coefficients other than zero, were included in the logistic regression model (Additional file 2: Figure S2).

**Table 2:** Demographic and clinical characteristics of patients in the derivation set distributed by 30-day mortality

Variable	Survivors *N* = 5226	Non-survivors *N* = 750	*p* value
Age (groups)			< 0.001
< 50	56.9	30.8	
50–65	22.8	18.1	
65–75	11.7	19.7	
> 75	8.6	31.3	
Sex (% male)	78.7	71.7	< 0.001
PRECOAG	11.3	30.4	< 0.001
Pre-hospital attention	75.6	79.7	0.014
Pre-hospital intubation	20.7	54.8	< 0.001
High Risk mechanism	34.5	63.1	< 0.001
Mechanism penetrant	6.0	4.3	0.054
PUPILS			< 0.001
Normal	93.8	52.7	
Unilateral mydriasis	5.3	19.6	
Bilateral mydriasis	0.9	27.7	
GCS ≤ 8 (%)	18.6	65.9	< 0.001
MAIS-Head	37.3	72.4	< 0.001
MAIS-Thorax	39.6	34.4	0.006
MAIS-Abdomen	12.1	11.5	0.601
MAIS-Ext Upper	2.8	1.7	0.082
MAIS-Ext Lower	18.2	14.4	0.011
MAIS-External	1.0	2.0	0.010
Haemodynamic failure	17.5	57.1	< 0.001
Respiratory failure	9.4	26.4	< 0.001
Coagulopathy	12.6	37.5	< 0.001
Mechanical ventilation	45.0	82.8	< 0.001
Massive haemorrhage	5.0	16.8	< 0.001

Values expressed as percentages

PRECOAG, prior treatment with antiplatelets or anticoagulants; GCS, Glasgow Coma Score; AIS, Abbreviated Injury Scale; MAIS, AIS ≥ 3; *p* value, calculated using chi-square test

Table 3 shows the final multivariate logistic regression (LR) model with internal validation (1000 bootstrap samples) with the 13 selected variables. *β*-coefficients multiplied by a shrinkage factor of 0.98.

**Table 3:** Multivariable analysis of factors associated with 30-day mortality

Variable	*β*-coefficients^a^	OR (95% CI)	*p* value	Points
Age groups				
< 50		Reference		0
50–65	0.598	1.83 (1.34–2.47)	< 0.001	1
65–75	1.239	4.78 (3.43–6.66)	< 0.001	2
> 75	2.198	10.35 (7.47–14.36)	< 0.001	4
PRECOAG	0.349	1.43 (1.10–1.85)	0.007	1
Pre-hospital intubation	0.336	1.41 (1.08–1.83)	0.012	1
High Risk mechanism	0.662	1.96 (1.56–2.47)	< 0.001	1
PUPILS				
Normal		Reference		0
Unilateral mydriasis	0.950	2.63 (1.97–3.51)	< 0.001	2
Bilateral mydriasis	3.217	26.38 (17.51–39.74)	< 0.001	6
GCS ≤ 8	0.841	2.35 (1.79–3.10)	< 0.001	2
MAIS-Head	0.495	1.66 (1.30–2.12)	< 0.001	1
MAIS-Thorax	− 0.271	0.76 (0.60–0.96)	0.026	− 1
Haemodynamic failure	1.148	3.21 (2.51–4.12)	< 0.001	2
Respiratory failure	0.708	2.06 (1.58–2.67)	< 0.001	1
Coagulopathy	0.567	1.78 (1.36–2.33)	< 0.001	1
Mechanical ventilation	0.580	1.80 (1.39–2.34)	< 0.001	1
Massive haemorrhage	0.452	1.58 (1.10–2.27)	< 0.001	1

Logistic regression. Derivation set (*n* = 5976)

PRECOAG, prior treatment with antiplatelets or anticoagulants; GCS, Glasgow Coma Score; AIS, Abbreviated Injury Scale; MAIS, AIS ≥ 3

^a^Regression coefficient multiplied with a shrinkage factor (bootstrapping procedure) of 0.98

The predicted probability of 30-day mortality was determined by the following equation:

Prob 30-day mortality = 1/(1 + exp (− *y*)), where *y* = 0.598 (Age 50–65) + 1.239 (Age 66–75) + 2.198 (Age > 75) + 0.349 (PRECOAG) + 0.336 (Pre-hospital intubation) + 0.662 (High risk mechanism) + 0.950 (unilateral mydriasis) + 3.217 (bilateral mydriasis) + 0.841 (Glasgow ≤ 8) + 0.495 (MAIS-Head) − 0.271 (MAIS-Thorax) + 1.148 (Haemodynamic failure) + 0.708 (Respiratory failure) + 0.567 (Coagulopathy) + 0.580 (Mechanical ventilation) + 0.452 (Massive haemorrhage) − 5.432. Predictor value is one when present and zero when absent.

Additional file 3 shows the stability of the model in the selection of variables and in the coefficients calculated using the comparison between the temporal validation and the use of random partitions.

### RETRASCORE model

**Fig. 1:**
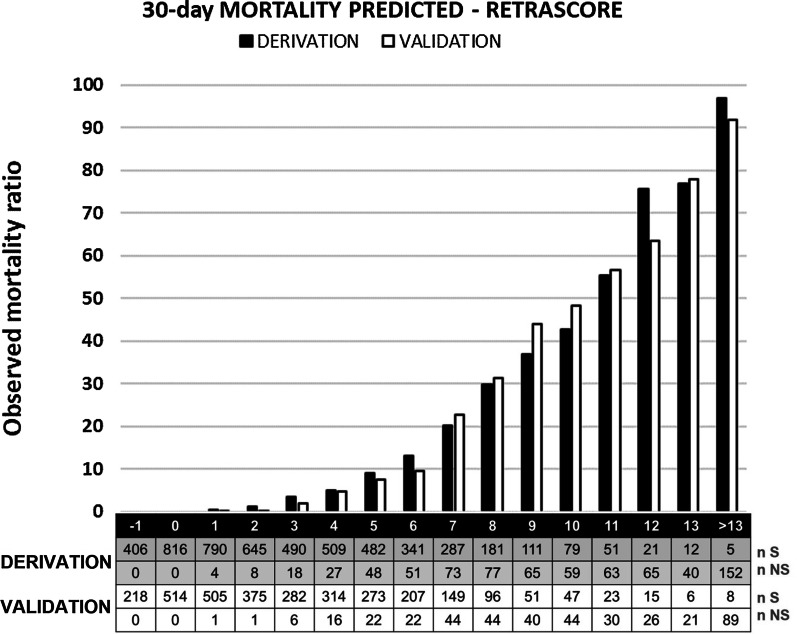
Probability of death according to the total score. Derivation and validation sets. S: survivors, NS: non-survivors

All factors associated with 30-day mortality in the LR model were used to develop the RETRASCORE model. Table 3 shows the points assigned to each factor. The sum of the different points constitutes the total score of the RETRASCORE. Figure 1 shows the probability of death, in the derivation and validation sets, according to the total score. Score values above 13 have been grouped by the limited number of records.

### Performance analysis of models

The LR model and the RETRASCORE models were analysed, and the TRISS model was used as a comparison. Additional file 2: Figures S3, S4 and S5 show the values of global performance (Brier score) in the three models, which was similar in the derivation and validation sets and amongst all of them; discrimination with the AUROC with higher values (from the DeLong test with *p* < 0.001) of the LR model and RETRASCORE (higher than 0.9) compared to the TRISS. The calibration is shown as a calibration curve in risk deciles and intercept and slope fit values, which were acceptable in the LR and RETRASCORE models and overestimated in the TRISS.

**Fig. 2:**
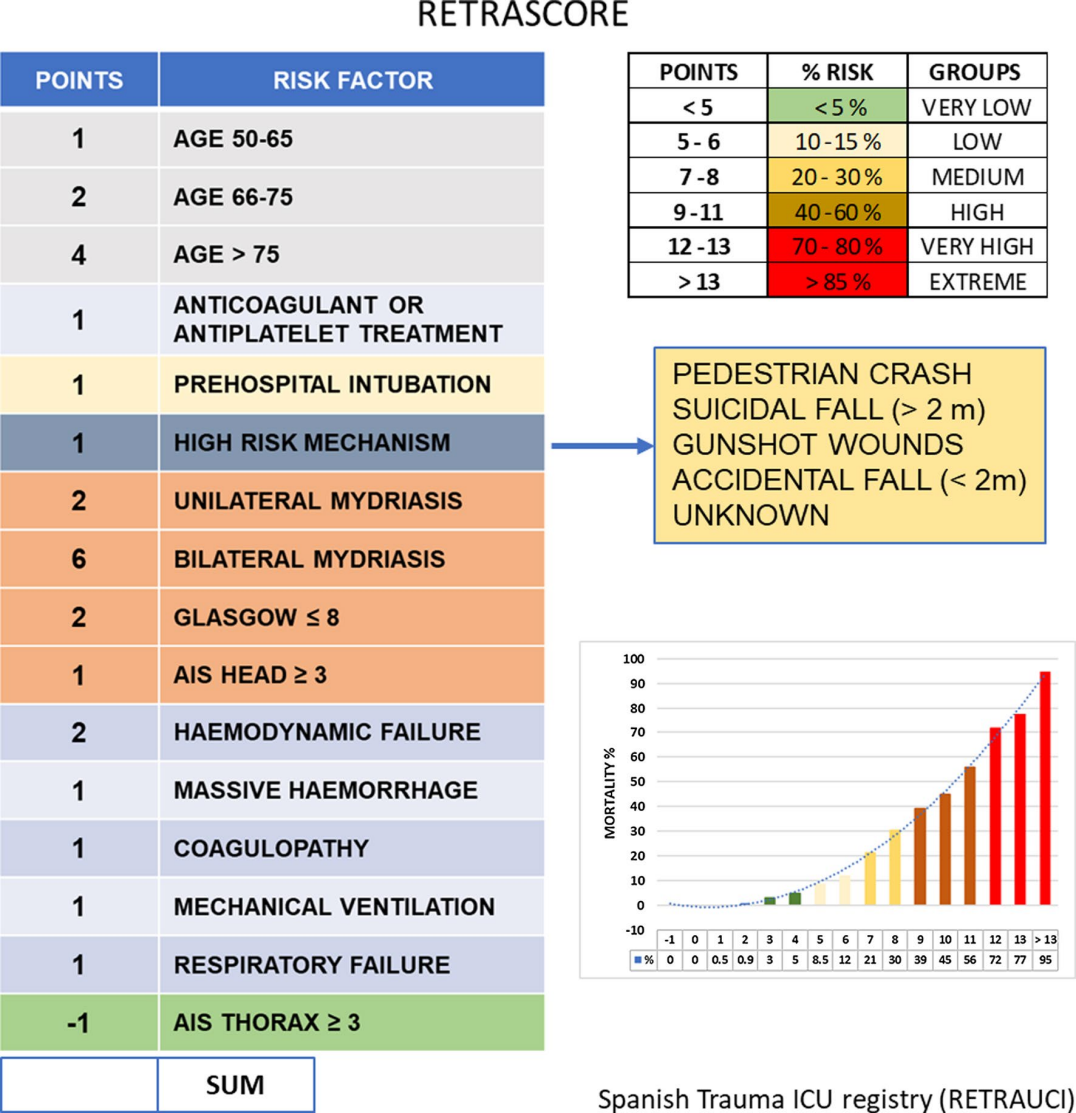
Summary sheet for the RETRASCORE model

**Fig. 3:**
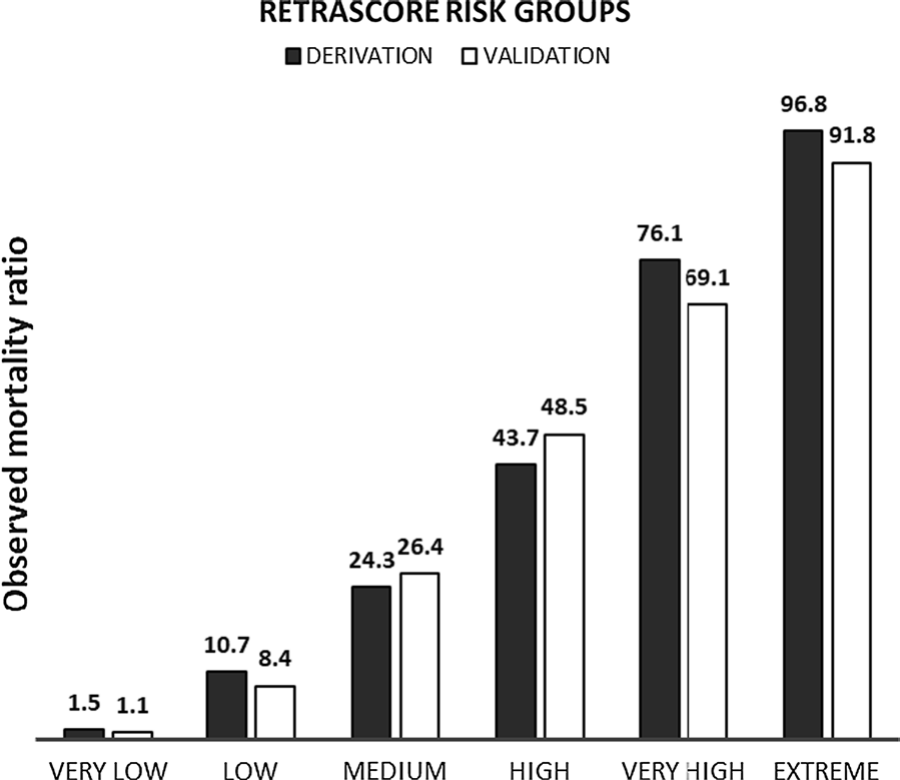
RETRASCORE risk categories in derivation and validation sets

Figure 2 shows the summary sheet for the RETRASCORE model. The predicted probability of death was grouped into six categories: very low (less than 5%) with less than 5 points, low (10–15%) with 5–6 points, medium (20–30%) with 7–8 points, high (40–60%) between 9 and 11 points, very high (70–80%) with 12–13 points, and extreme (higher than 90%) with > 13 points. Figure 3 shows these separate probability categories in the derivation and validation sets.

## Discussion

The appropriate use of trauma scoring is of paramount importance in terms of outcome adjustment and benchmarking of the care provided [20]. The ideal score for stratifying the risk of death in the trauma ICU should be performed early, must be easy-to-calculate and include specific factors derived from the intensive care management of critical trauma patients. However, classical trauma-specific scoring systems (ISS, RTS, TRISS) are still used with a poor calibration and discrimination capacity, and their performance is lower than that obtained by general ICU scores where anatomical injuries caused by trauma are not considered [21, 22], raising the need of specific trauma ICU scores that take into consideration early anatomic consideration but also pre-hospital care, physiological derangements, organ failures and treatments provided. We believe that the model presented meets these expectations [23].

Different types of variables were finally included in our model, according to its relationship with mortality in conventional models.

Non-modifiable factors depending on the patient were age and the prior treatment of antiplatelet/anticoagulant therapy. Age is a critical factor in the outcomes of trauma patients, as mortality increases with age [8, 24]. On the other side, prior treatment with antiplatelets/anticoagulants contributes to the size and burden of traumatic injuries, especially in patients with traumatic brain injury (TBI) [11].

In our model, two variables related to the pre-hospital care were included, the type of attention with trained medical attention specialized in advanced resuscitation techniques and the need of pre-hospital intubation [25, 26]. These factors are closely related to the quality of a mature trauma system in each country. We also considered type and intentionality of trauma. We grouped different mechanisms into a single one variable associated with high mortality including pedestrian falls, suicidal high-energy falls, firearms injuries and unknown mechanisms [27, 28]. These are well-known mechanisms associated with higher mortality. For the purpose of an external validation of our score, it would be especially important to check how this variable performs.

TBI is the leading cause of mortality in the trauma ICU in our environment. Therefore, it is not surprising that three neurological factors were finally included in our model. All of them are well-known clinical factors related to outcomes: pupillary reactivity, which is easy to determine and carries an important weight in TBI [8, 29], GCS ≤ 8 and the anatomical burden of head injury evaluated by the AIS [8].

Additionally, haemodynamic instability, respiratory failure and trauma-induced coagulopathy in patients with critical bleeding are associated with a worst prognosis and were also present in our model [13, 14, 30, 31].

The need for MV is an indicator of severity in critical trauma patients. It does not only potentially express respiratory failure, but also neurological or haemodynamic involvement or the need of procedures that require sedation.

We observed that severe chest trauma was a protective factor of trauma ICU patients. This intriguing result merits further investigation since the real contribution of chest trauma to the mortality of severe trauma patients is still a matter of debate [32]. Approximately 600 patients were admitted to the participating ICUs with severe chest trauma and without other major injuries, and most of these patients received pain control, had early physiotherapy and had a good outcome. Likely, this is the reason why severe chest trauma received a score of − 1 in the RETRASCORE model.

Taking together these variables, our easy-to-calculate and specific trauma ICU score achieves an excellent performance in terms of mortality prediction with an AUROC of almost 0.93 in the validation dataset. This is very close to the astonishing predictive ability of the RISC-II updated score, but ours is specific to the trauma ICU patients and is easier to use since it does not include data on pre-trauma ASA status, base deficit, haemoglobin and cardiopulmonary resuscitation [8]. Instead, it uses common clinical conditions such as respiratory failure or trauma-induced coagulopathy that reflect the physiological response to trauma based on predefined definitions. In this line, our score should be also confronted with general ICU scores. Magee et al. recently compared general ICU scores with traditional trauma scores and observed an improved performance of general ICU scores evaluating physiological derangements in the initial 24 h over anatomical scores [20]. Due to the increasing age of trauma populations who carry on additional comorbidities, we believe that scores like ours combining anatomical injuries and physiological derangements in the initial 24 h will improve our early prediction ability, especially in different subgroups of trauma patients [21].

We expect our score to be further externally validated in different databases allowing additional comparison with general ICU scores.

Our study presents some limitations:

First, the most important limitation is the lack of an external validation. This must be performed before the score can be applied in daily clinical practice. Second, the inclusion criteria of being admitted to the participating ICUs may not appropriately reflect the critical trauma population due to differences in admission criteria and bed and staffing availability. Third, unless patients were managed following the *Advanced Trauma Life Support* principles, we cannot rule out deviations so this could affect patients’ management and therefore outcomes [22]. Fourth, we have only used the TRISS as a comparison model; RETRASCORE should be compared with other scores, both specific to trauma patients and of a general type. Fifth, if a random partition had been made of the derivation and validation groups, no differences would have been found between the sets; we chose a temporary partition since we had a sufficiently large sample that could provide us with a temporal validation [10]. Finally, machine learning techniques have been used in our environment to predict outcome [33], but in this study, our objective was to develop a simple and early, easy-to-calculate and specific trauma ICU score rather than using complex methodologies.

## Conclusions

In conclusion, the newly developed RETRASCORE is an early, easy-to-calculate and specific score to predict in-hospital mortality in trauma ICU patients. This is the first trauma score specifically designed for the trauma ICU population. Although it has achieved adequate internal validation, it must be externally validated.

## Supplementary Information

**Additional file 1:** Screenshots of the web application and the list of variables collected in RETRAUCI.

**Additional file 2: Figure S2-1.** Flow diagram for selection of patients. **Figure S2-2:** Selection of variables using LASSO (Least Absolute Shrinkage and Selection Operator) binary logistic regression model. **Figure S2-3**. Performance evaluation of the logistic regression (LR) model. **Figure S2-4**. Performance evaluation of the TRISS model. **Figure S2-5**. Performance evaluation of the RETRASCORE model.

**Additional file 3:** Comparative analysis between temporal and random partitions in the selection of variables and calculated coefficients.

## Data Availability

The data are available for other investigators’ use under reasonable request. Please contact the corresponding author for access requests.

## Abbreviations

ISS: Injury Severity Score
TRISS: Trauma and Injury Score
TR-DGU: Trauma Register DGU
RISC: Revised Injury Severity Classification
AUROC: Area under the receiver operating characteristic curve
ICU: Intensive care unit
APACHE II: Acute Physiology and Chronic Health Evaluation
SAPS II: Simplified Acute Physiologic Score
MPM II: Mortality probability models
RETRAUCI: Spanish Trauma ICU Registry
TRIPOD: Transparency Reporting of a multivariable prediction model for Individual Prognosis or Diagnosis
PRECOAG: Prior coagulation alteration
GCS: Glasgow Coma Score
AIS: Abbreviated Injury Scale
MAIS: Major involvement with AIS of 3 or higher
LOS: Length of stay
RETRASCORE: Simple score made with the RETRAUCI database
LR: Logistic regression model
CI: Confidence interval
LASSO: Least Absolute Shrinkage and Selection Operator
TBI: Traumatic brain injury
