# Supplementary material for: Development of a new score for early mortality prediction in trauma ICU patients: RETRASCORE
Source: Crit Care. 2021 Dec 7;25:420. doi: 10.1186/s13054-021-03845-6 (PMC8650319; doi:10.1186/s13054-021-03845-6)
Supplement: Supplementary file 1 — Additional file 1: Screenshots of the web application and the list of variables collected in RETRAUCI. [file 13054_2021_3845_MOESM1_ESM.docx]

**Additional file 1**

**Development of a new score for early mortality prediction in trauma ICU patients. RETRASCORE**

Luis Serviá^1^, Juan Antonio Llompart-Pou^2^, Mario Chico-Fernández^3^, Neus Montserrat^1^, Mariona Badia^1^, Jesús Abelardo Barea-Mendoza^3^, María Ángeles Ballesteros-Sanz^4^, and Javier Trujillano^1*^

On behalf of the Neurointensive Care and Trauma Working Group of the Spanish Society of Intensive Care Medicine (SEMICYUC)

1. Servei de Medicina Intensiva. Hospital Universitari Arnau de Vilanova. Universitat de Lleida. IRBLleida. Lleida.
2. Servei de Medicina Intensiva. Hospital Universitari Son Espases. Institut d’Investigació Sanitària Illes Balears (IdISBa). Palma de Mallorca.
3. UCI de Trauma y Emergencias. Servicio de Medicina Intensiva. Hospital Universitario 12 de Octubre. Madrid.
4. Servicio de Medicina Intensiva. Hospital Universitario Marqués de Valdecilla. Santander.

[lserviag@gmail.com](mailto:lserviag@gmail.com)

[juanantonio.llompart@ssib.es](mailto:juanantonio.llompart@ssib.es)

[murgchico@yahoo.es](mailto:murgchico@yahoo.es)

[neus-montserrat@hotmail.es](mailto:neus-montserrat@hotmail.es)

[mbadia26@gmail.com](mailto:mbadia26@gmail.com)

[elbarea@gmail.com](mailto:elbarea@gmail.com)

[gelesballesteros@yahoo.com](mailto:gelesballesteros@yahoo.com)

jtruji@cmb.udl.es

(*)Correspondence:

Javier Trujillano

Intensive Care Unit

Hospital Universitario Arnau de Vilanova

Avda Rovira Roure 80

25198 Lleida (Spain)

e-mail: [jtruji@cmb.udl.es](mailto:marionabadia@wanadoo.es)

Tel: +34-973705248

Fax: +34-973221055

**Additional file 1:**

This additional document shows the screenshots of the web application of the RETRAUCI project database.

The list of variables has been added. They have been classified as type of variables: (N) - Numerical, (C) - Categorical, (D) - Date and (NA) - Not available.

The definition of the variables used to create the model has been defined in the Methods section.

**Figure S1-1:** RETRAUCI database home screen.

**Figure S1-2:** Variable input screen of the general and epidemiological data section.

**Figure S1-3:** Variable input screen of the traumatic injury coding section.

**Figure S1-4:** Variables input screen of the gravity scores calculation section

**Figure S1-5:** Variable input screen of the performed procedures section.

**Figure S1-6:** Variables input screen of the evolutionary complications section.

**Figure S1-7:** Variable entry screen for the section on the evolution of traumatic patients admitted to the ICU.
