# Supplementary material for: Development of a new score for early mortality prediction in trauma ICU patients: RETRASCORE
Source: Crit Care. 2021 Dec 7;25:420. doi: 10.1186/s13054-021-03845-6 (PMC8650319; doi:10.1186/s13054-021-03845-6)
Supplement: Supplementary file 2 — Additional file 2: Figure S2-1. Flow diagram for selection of patients. Figure S2-2: Selection of variables using LASSO (Least Absolute Shrinkage and Selection Operator) binary logistic regression model. Figure S2-3. Performance evaluation of the logistic regression (LR) model. Figure S2-4. Performance evaluation of the TRISS model. Figure S2-5. Performance evaluation of the RETRASCORE model. [file 13054_2021_3845_MOESM2_ESM.docx]

**Additional file 2**

**Development of a new score for early mortality prediction in trauma ICU patients. RETRASCORE**

Luis Serviá^1^, Juan Antonio Llompart-Pou^2^, Mario Chico-Fernández^3^, Neus Montserrat^1^, Mariona Badia^1^, Jesús Abelardo Barea-Mendoza^3^, María Ángeles Ballesteros-Sanz^4^, and Javier Trujillano^1*^

On behalf of the Neurointensive Care and Trauma Working Group of the Spanish Society of Intensive Care Medicine (SEMICYUC)

1. Servei de Medicina Intensiva. Hospital Universitari Arnau de Vilanova. Universitat de Lleida. IRBLleida. Lleida.
2. Servei de Medicina Intensiva. Hospital Universitari Son Espases. Institut d’Investigació Sanitària Illes Balears (IdISBa). Palma de Mallorca.
3. UCI de Trauma y Emergencias. Servicio de Medicina Intensiva. Hospital Universitario 12 de Octubre. Madrid.
4. Servicio de Medicina Intensiva. Hospital Universitario Marqués de Valdecilla. Santander.

[lserviag@gmail.com](mailto:lserviag@gmail.com)

[juanantonio.llompart@ssib.es](mailto:juanantonio.llompart@ssib.es)

[murgchico@yahoo.es](mailto:murgchico@yahoo.es)

[neus-montserrat@hotmail.es](mailto:neus-montserrat@hotmail.es)

[mbadia26@gmail.com](mailto:mbadia26@gmail.com)

[elbarea@gmail.com](mailto:elbarea@gmail.com)

[gelesballesteros@yahoo.com](mailto:gelesballesteros@yahoo.com)

jtruji@cmb.udl.es

(*)Correspondence:

Javier Trujillano

Intensive Care Unit

Hospital Universitario Arnau de Vilanova

Avda Rovira Roure 80

25198 Lleida (Spain)

e-mail: [jtruji@cmb.udl.es](mailto:marionabadia@wanadoo.es)

Tel: +34-973705248

Fax: +34-973221055

**Additional file 2: Figure S2-11:** **Flow diagram for selection of patients.**


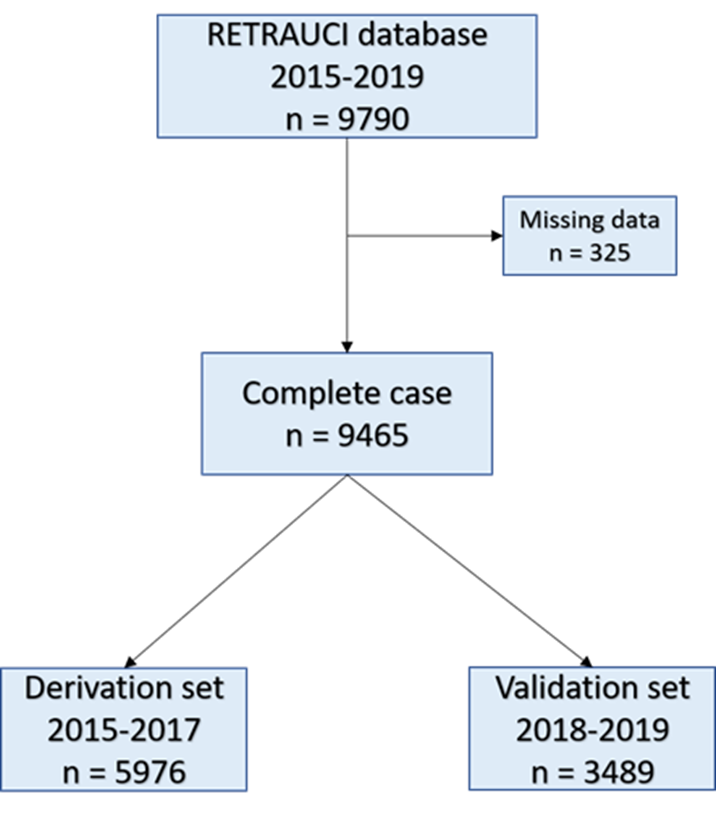


**Additional file 2: Figure S2-2:** **Selection of variables using LASSO (least absolute shrinkage and selection operator) binary logistic regression model.**

(A) Optimal lambda selection in the LASSO model using 10-fold-cross validation, a partial likelihood deviance curve was plotted versus Log lambda. Dotted lines were drawn at the optimal values. (B) LASSO coefficients profiles of the 20 variable candidates. Dotted line indicates the best lambda value that determines an optimal model with 13 variables with coefficients other than zero (lambda = 0.0068, log(lambda) = -4.98).


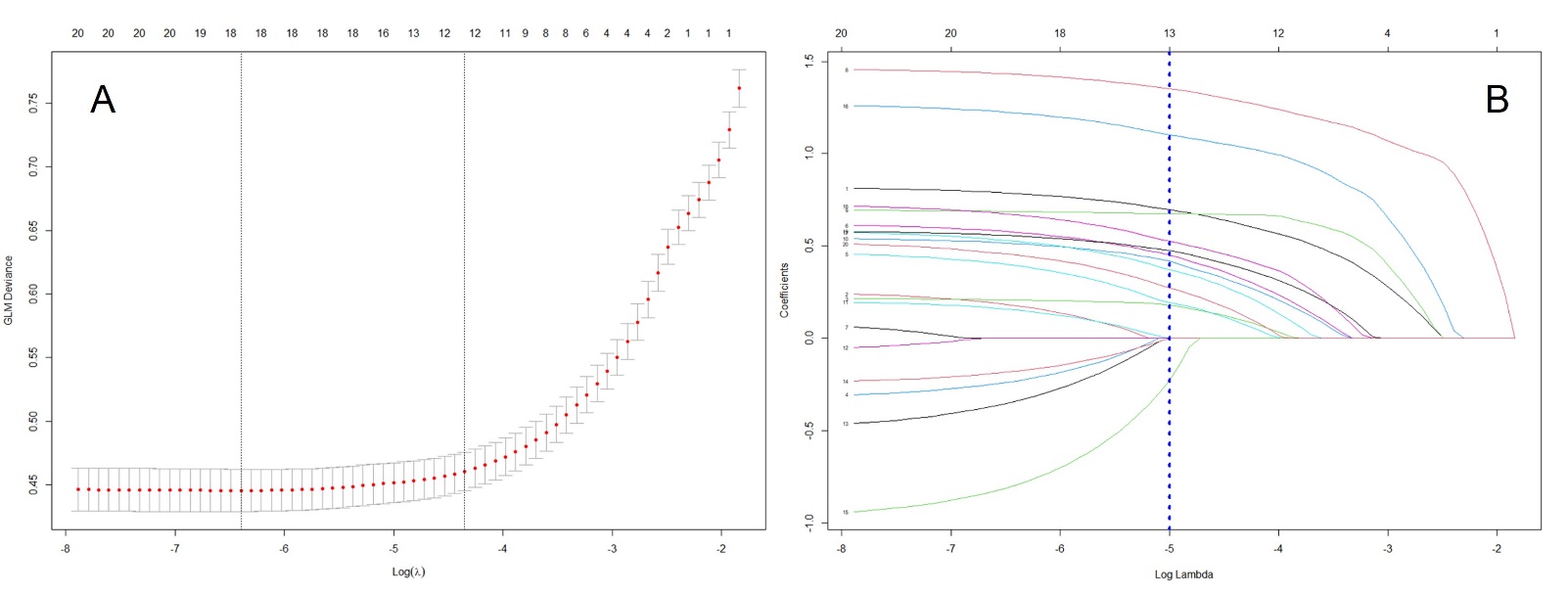


In the process of selecting the variables to be included in the definitive model, the LASSO algorithm helps us to determine within the 20 candidate variables those with the greatest weight, which are those that show, once regularized, coefficients other than a value of zero.

In figure A the algorithm defines the best number of variables to include in a range between 12 and 18. And, in figure B the 13 variables that should be included in the definitive model are identified.

**Additional file 2: Figure S2-3:** **Performance evaluation of the Logistic Regression (LR) model.**


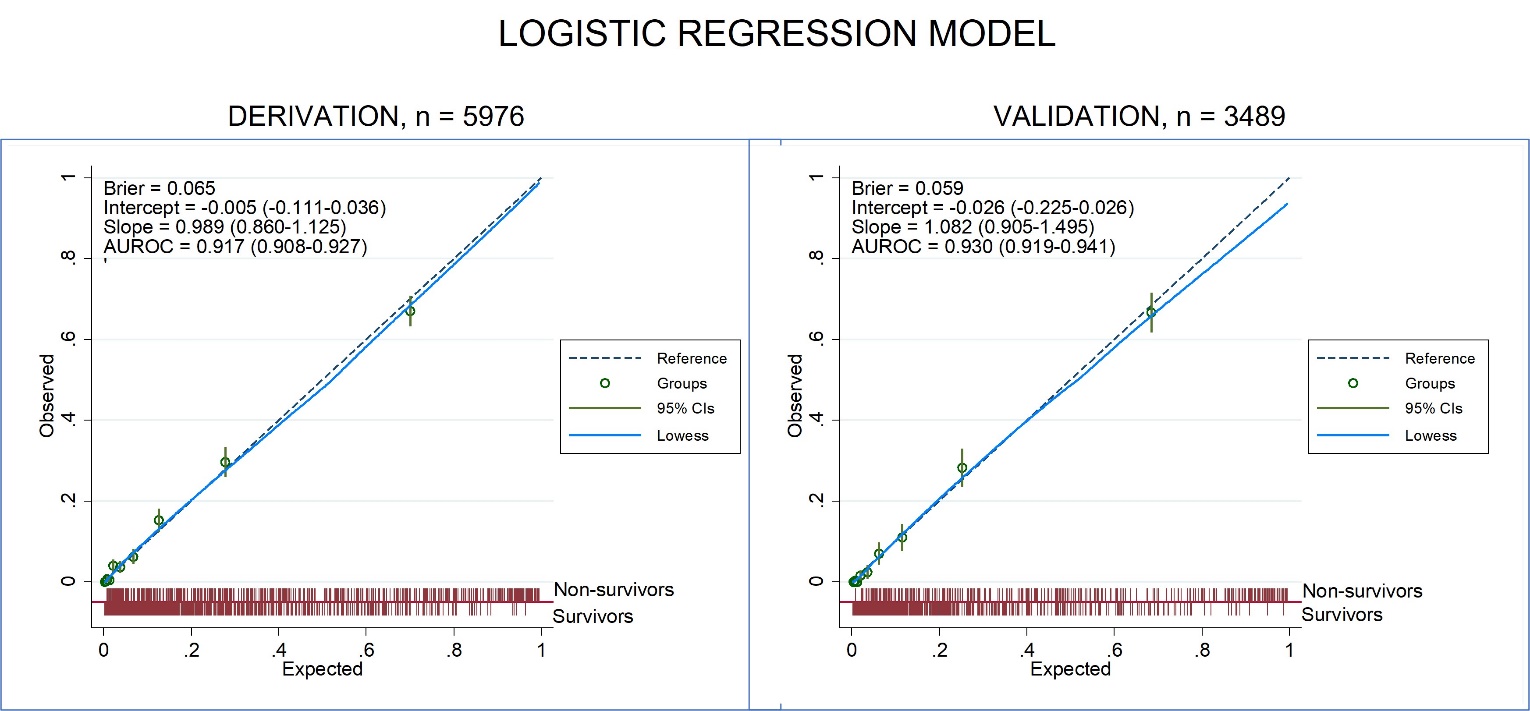


**Additional file 2: Figure S2-4:** **Performance evaluation of the TRISS model.**


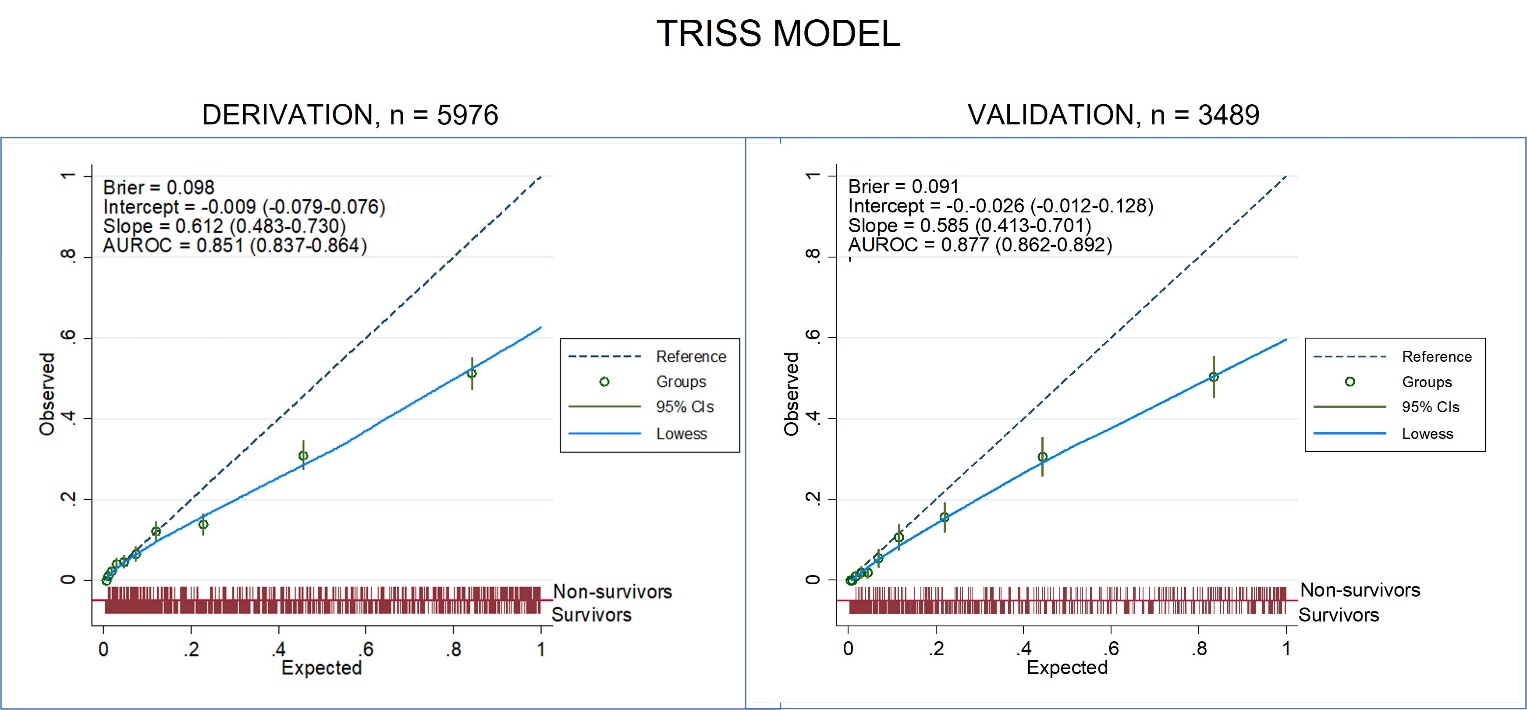


**Additional file 2: Figure S2-5:** **Performance evaluation of the RETRASCORE model.**


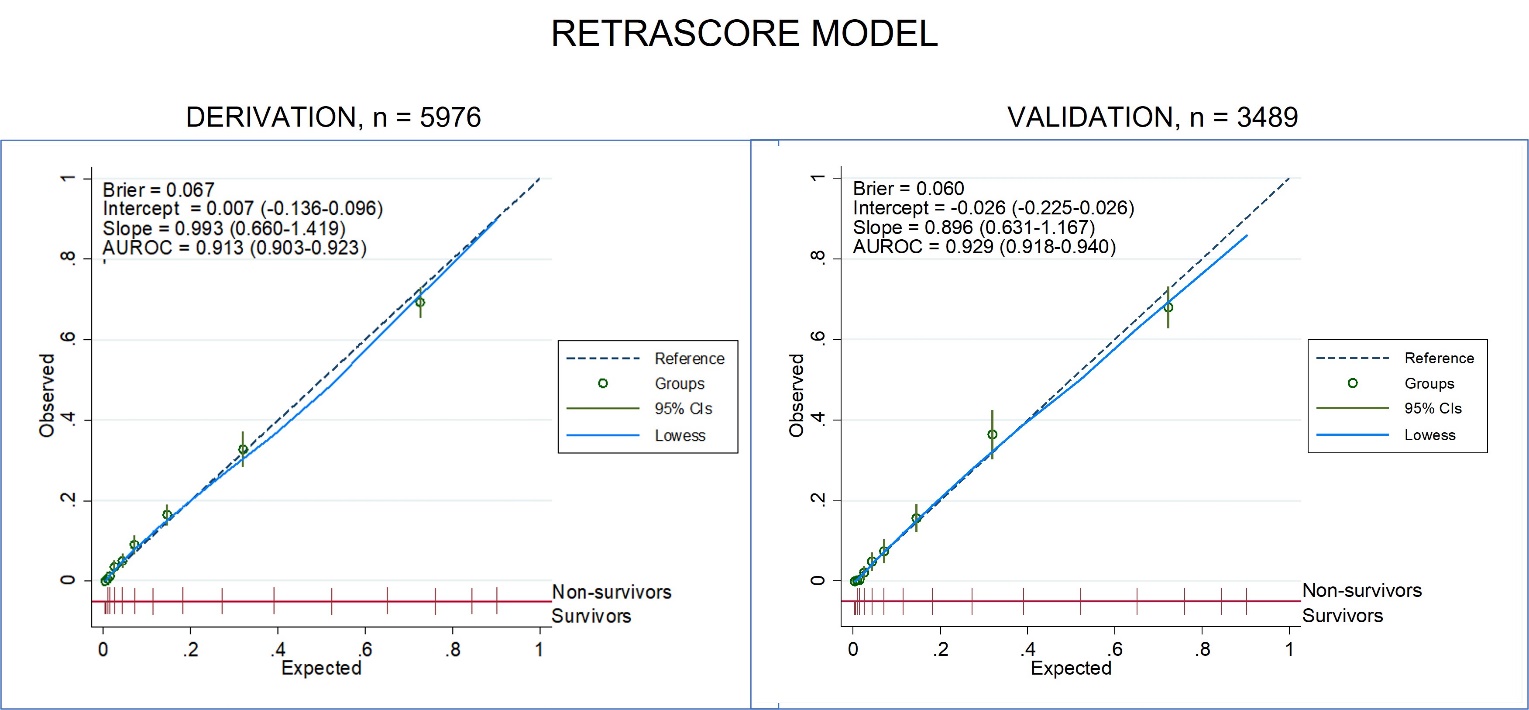


The basic interpretation of the evaluated parameters. Optimal values: global assessment with Brier of 0, discrimination with AUCROC of 1, and calibration with Intercept of 0 and Slope of 1.

The LR model and the RETRASCORE models were analysed and the TRISS model was used as a comparison. Additional file 1: Figure S3, S4 and S5 show the values of global performance (Brier score) in the three models, which was similar in the derivation and validation sets and amongst all of them; discrimination with the AUROC with higher values (from the DeLong test with p <0.001) of the LR model and RETRASCORE (higher than 0.9) compared to the TRISS. The calibration is shown as a calibration curve in risk deciles and intercept and slope fit values, which were acceptable in the LR and RETRASCORE models and showed an overestimation in the TRISS.
