# Supplementary material for: Development of a new score for early mortality prediction in trauma ICU patients: RETRASCORE
Source: Crit Care. 2021 Dec 7;25:420. doi: 10.1186/s13054-021-03845-6 (PMC8650319; doi:10.1186/s13054-021-03845-6)
Supplement: Supplementary file 3 — Additional file 3: Comparative analysis between temporal and random partitions in the selection of variables and calculated coefficients. [file 13054_2021_3845_MOESM3_ESM.docx]

**Additional file 3**

**Development of a new score for early mortality prediction in trauma ICU patients. RETRASCORE**

Luis Serviá^1^, Juan Antonio Llompart-Pou^2^, Mario Chico-Fernández^3^, Neus Montserrat^1^, Mariona Badia^1^, Jesús Abelardo Barea-Mendoza^3^, María Ángeles Ballesteros-Sanz^4^, and Javier Trujillano^1*^

On behalf of the Neurointensive Care and Trauma Working Group of the Spanish Society of Intensive Care Medicine (SEMICYUC)

1. Servei de Medicina Intensiva. Hospital Universitari Arnau de Vilanova. Universitat de Lleida. IRBLleida. Lleida.
2. Servei de Medicina Intensiva. Hospital Universitari Son Espases. Institut d’Investigació Sanitària Illes Balears (IdISBa). Palma de Mallorca.
3. UCI de Trauma y Emergencias. Servicio de Medicina Intensiva. Hospital Universitario 12 de Octubre. Madrid.
4. Servicio de Medicina Intensiva. Hospital Universitario Marqués de Valdecilla. Santander.

[lserviag@gmail.com](mailto:lserviag@gmail.com)

[juanantonio.llompart@ssib.es](mailto:juanantonio.llompart@ssib.es)

[murgchico@yahoo.es](mailto:murgchico@yahoo.es)

[neus-montserrat@hotmail.es](mailto:neus-montserrat@hotmail.es)

[mbadia26@gmail.com](mailto:mbadia26@gmail.com)

[elbarea@gmail.com](mailto:elbarea@gmail.com)

[gelesballesteros@yahoo.com](mailto:gelesballesteros@yahoo.com)

jtruji@cmb.udl.es

(*)Correspondence:

Javier Trujillano

Intensive Care Unit

Hospital Universitario Arnau de Vilanova

Avda Rovira Roure 80

25198 Lleida (Spain)

e-mail: [jtruji@cmb.udl.es](mailto:marionabadia@wanadoo.es)

Tel: +34-973705248

Fax: +34-973221055

**Additional file 3:** Comparative analysis between temporal and random partition in the selection of variables and calculated coefficients.

As a complementary analytical work, a comparison has been made between the temporal and the random partition. The objective of this work is to confirm the stability in the selection of variables of the model used and in the calculation of the logistic regression coefficients that have been used for the creation of the RETRASCORE.

100 random sets were obtained with 5976 patients each (out of a total of 9465). We have kept the same number as used for the temporal validation.

**Table S3-1.** Selection percentage of each variable in the 100 randomized partition sets.

| **Variable** | **SELECTED** | | |
| --- | --- | --- | --- |
| **Age (groups)** | |  |  |
| **< 50** | | 100 |  |
| **50 - 65** | | 100 |  |
| **65 - 75** | | 100 |  |
| **> 75** | | 100 |  |
| **Sex (% male)** | | 4 |  |
| **PRECOAG** | | 92 |  |
|  | |  |  |
| **Pre-hospital attention** | | 4 |  |
| **Pre-hospital intubation** | | 93 |  |
|  | |  |  |
| **High Risk mechanism** | | 100 |  |
| **Mechanism penetrant** | | 0 |  |
|  | |  |  |
| **PUPILS** | |  |  |
| **Normal** | |  |  |
| **Unilateral mydriasis** | | 100 |  |
| **Bilateral mydriasis** | | 100 |  |
|  | |  |  |
| **GCS ≤ 8 (%)** | | 100 |  |
|  | |  |  |
| **MAIS-Head** | | 100 |  |
| **MAIS-Thorax** | | 91 |  |
| **MAIS-Abdomen** | | 0 |  |
| **MAIS-Ext Upper** | | 0 |  |
| **MAIS-Ext Lower** | | 9 |  |
| **MAIS-External** | | 0 |  |
|  | |  |  |
| **Haemodynamic failure** | | 100 |  |
| **Respiratory failure** | | 100 |  |
| **Coagulopathy** | | 100 |  |
|  | |  |  |
| **Mechanical ventilation** | | 100 |  |
| **Massive haemorrhage** | | 100 |  |

SELECTED: Percentage of times the variable has been selected from the total of 100 random partitions. PRECOAG: Prior treatment with antiplatelets or anticoagulants; GCS: Glasgow coma score; AIS: Abbreviated Injury Scale; MAIS: AIS ≥3.

First, the LASSO variable selection algorithm was used to obtain the selected variables in each of the 100 sets. The table S3-1shows the number of times that each variable in the group of candidates was selected. In 82 of the sets, the same selection of variables coincided as that obtained in the temporal validation.

Afterwards, the logistic regression coefficients were calculated for each of the 82 random sets that presented the same selection of variables as that used in the temporal validation. The following table shows the intervals (95%) of the calculated mean values, ​​ being compared with the values used in the temporal validation. It is observed that in all cases the values used in the temporal validation are included within the intervals obtained in the random sets.

**Table S3-2.** Comparison of coefficients obtained by temporal validation against those calculated for 100 random partitions.

|  | **Temporal validation** | **Randomly splitting (CI 95%)** |
| --- | --- | --- |
| **Variable** | **β-coefficients** | **β-coefficients** |
| **Age groups** |  |  |
| **< 50** |  |  |
| **50 - 65** | 0.598 | 0.582 – 1.025 |
| 65 - 75 | 1.239 | 1.188 – 1.779 |
| **> 75** | 2.198 | 2.117 – 2.704 |
|  |  |  |
| **PRECOAG** | 0.349 | 0.183 – 0.489 |
|  |  |  |
| **Pre-hospital intubation** | 0.336 | 0.167 – 0.500 |
|  |  |  |
| **High Risk mechanism** | 0.662 | 0.490 – 0.810 |
|  |  |  |
| **PUPILS** |  |  |
| **Normal** |  |  |
| **Unilateral mydriasis** | 0.950 | 0.882 – 1.226 |
| **Bilateral mydriasis** | 3.217 | 2.939 – 3.418 |
|  |  |  |
| **GCS ≤ 8** | 0.841 | 0.731 – 1.037 |
| **MAIS-Head** | 0.495 | 0.428 – 0.717 |
| **MAIS-Thorax** | -0.271 | -0.302 – -0.092 |
|  |  |  |
| **Haemodynamic failure** | 1.148 | 0.970 – 1.316 |
| **Respiratory failure** | 0.708 | 0.511 – 0.861 |
| **Coagulopathy** | 0.567 | 0.439 – 0.830 |
|  |  |  |
| **Mechanical ventilation** | 0.580 | 0.511 – 0.861 |
| **Massive haemorrhage** | 0.452 | 0.345 – 1.041 |

PRECOAG: Prior treatment with antiplatelets or anticoagulants; GCS: Glasgow coma score; AIS: Abbreviated Injury Scale; MAIS: AIS ≥3.
